# PKC-Dependent GlyT1 Ubiquitination Occurs Independent of Phosphorylation: Inespecificity in Lysine Selection for Ubiquitination

**DOI:** 10.1371/journal.pone.0138897

**Published:** 2015-09-29

**Authors:** Susana P. Barrera, Vicente Castrejon-Tellez, Margarita Trinidad, Elisa Robles-Escajeda, Javier Vargas-Medrano, Armando Varela-Ramirez, Manuel Miranda

**Affiliations:** Department of Biological Sciences and Border Biomedical Research Center, University of Texas at El Paso, El Paso, TX, 79968, United States of America; Institut Curie, FRANCE

## Abstract

Neurotransmitter transporter ubiquitination is emerging as the main mechanism for endocytosis and sorting of cargo into lysosomes. In this study, we demonstrate PKC-dependent ubiquitination of three different isoforms of the glycine transporter 1 (GlyT1). Incubation of cells expressing transporter with the PKC activator phorbol ester induced a dramatic, time-dependent increase in GlyT1 ubiquitination, followed by accumulation of GlyT1 in EEA1 positive early endosomes. This occurred via a mechanism that was abolished by inhibition of PKC. GlyT1 endocytosis was confirmed in both retinal sections and primary cultures of mouse amacrine neurons. Replacement of only all lysines in the *N*-and *C*-termini to arginines prevented ubiquitination and endocytosis, displaying redundancy in the mechanism of ubiquitination. Interestingly, a 40–50% reduction in glycine uptake was detected in phorbol-ester stimulated cells expressing the WT-GlyT1, whereas no significant change was for the mutant protein, demonstrating that endocytosis participates in the reduction of uptake. Consistent with previous findings for the dopamine transporter DAT, ubiquitination of GlyT1 tails functions as sorting signal to deliver transporter into the lysosome and removal of ubiquitination sites dramatically attenuated the rate of GlyT1 degradation. Finally, we showed for the first time that PKC-dependent GlyT1 phosphorylation was not affected by removal of ubiquitination sites, suggesting separate PKC-dependent signaling events for these posttranslational modifications.

## Introduction

The neurotransmitter glycine is responsible for the regulation of several essential functions in the central and peripheral nervous system including motor and sensory signals. It plays a dual role in the CNS and participates at both inhibitory and excitatory synapses. At inhibitory synapses, glycine released at the synaptic cleft binds to post-synaptic strychnine-sensitive glycine receptors, resulting in chloride permeability and hyperpolarization of the post-synaptic neuron. In addition, glycine functions as an essential co-agonist with glutamate at NMDA-receptor containing synapses, where binding and further activation of the receptor allows Ca^++^ influx and propagation of neurotransmission. Termination of neurotransmission at glycinergic synapses is achieved by rapid re-uptake of glycine into the presynaptic neurons and surrounding glial cells by two glycine transporters, GlyT1 and GlyT2. At NMDA-containing synapse, GlyT1 is in charge of glycine clearance. Several pieces of experimental evidence support the essential role of GlyT1 in the regulation of NMDA receptor activity by maintaining a sub-saturating concentration of glycine at the glutamatergic synaptic cleft [1,2,3,4].

**Fig 1:**
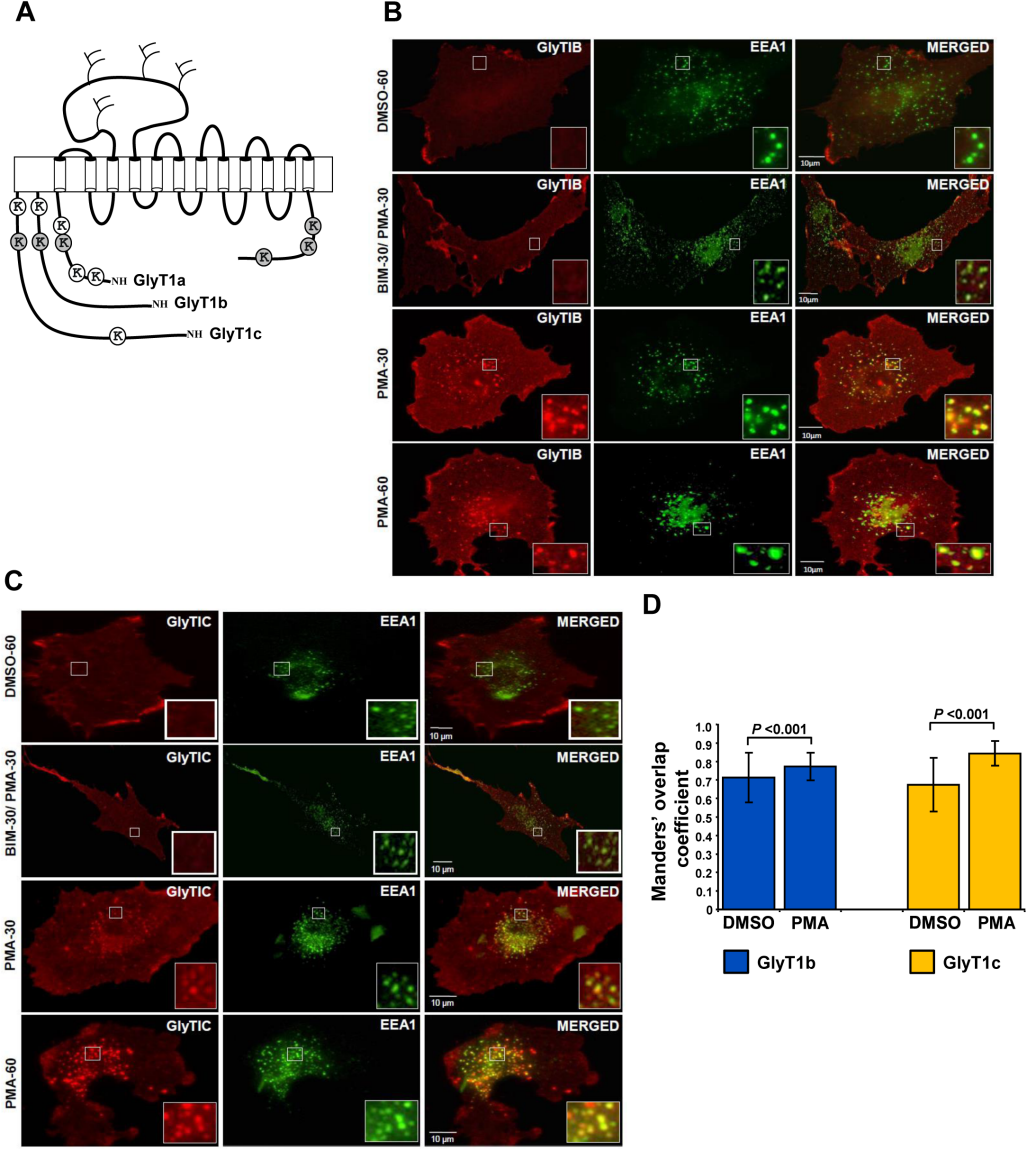
Schematic representation of the predicted topology of GlyT1 isoforms and PKC- induced endocytosis of GlyT1. **A)** The twelve membrane-spanning segments are depicted by cylinders, and intracellular *N* and *C* termini, loops and extracellular glycosylation sites by solid lines. The position of lysine residues in the three different *N*-terminal splice variants are presented by beads (GlyT1a, 1b and 1c). Conserved lysines are highlighted by gray beads. **B)** PAE cells stably expressing FH-GlyT1b and **C)** PAE cells stably expressing FH-GlyT1c were incubated with DMSO or PMA for 30–60 min at 37°C, fixed and immunostained with anti-GlyT1 and anti-EAA1 antibodies followed by incubation with a CY-3 and Alexa 488 labeled secondary antibodies. Images were selected to represent the cell population and acquired through YFP (green) and CY3 (red) filter channels. Single optical sections through the middle of the cells are shown. ‘Yellow’ in the merged images signifies co-localization of CY3 (GlyT1) and YFP (EEA1). D) Co-localization was quantified in pixel by pixel bases from images obtained by confocal microscopy using the Mander’s overlap coefficient of merged images. A value of 1 represents 100% co-localization of both fluorescence signals in 15 randomly selected endosomes, whereas a zero value denotes complete absence of co-localization. *p* values were determine by student’s *t*-test. *Scale bars*, 10 μm.

The transporters GlyT1 and GlyT2 belong to the SLC6 family of Na^+^, Cl^-^-dependent neurotransmitter transporters, which include the monoamine (DAT, SERT and NET), GABA and proline carriers. Structurally, the GlyTs are polytopic membrane proteins embedded in the plasma membrane by 12 transmembrane-spanning domains, a large extracellular loop containing 3–4 glycosylation sites and intracellular amino- and carboxyl-terminal tails carrying predicted sites for posttranslational modifications (Fig 1A). Although GlyTs share about 50% sequence identity, they differ in their tissue-distribution pattern. GlyT2 is found exclusively in neurons from regions enriched in synapses that contain glycine receptors (glycinergic synapses), predominantly the brain stem, cerebellum and spinal cord. In contrast, it has been proposed that GlyT1 is expressed in glial cells surrounding glycinergic synapses, in addition to glutamatergic neurons and glial cells from the hippocampus, cortex and other subcortical regions such as the thalamus [5,6]. Three different amino-terminal GlyT1 splice variants, encoded by the same gene, have been identified (GlyT1a, b and c) that differ in the length of the amino terminal tail [7]. The GlyT1a variant represents the shortest isoform followed by GlyT1b and GlyT1c (Fig 1A).

As with all neurotransmitter transporters, the activities of the GlyTs are dependent on their localization to the plasma membrane, and internalization results in a reduction in the uptake capacity. However, the mechanisms by which their localization and activities are regulated have not been examined in detail until now. For most members of the SLC6 family, both of these properties are tightly-connected to a signaling pathway that involves *P*rotein *K*inase *C* (PKC). For several SLC6 family members, including GlyT1, PKC activation by phorbol ester (PMA) diminishes neurotransmitter maximal transport capacity and increases both transporter phosphorylation and interaction with the SNARE protein syntaxin 1A [8,9,10]. These properties, which result in inhibition of transporter activity, have been attributed to increased endocytosis. Additional evidence also supports the role of other signaling molecules in the regulation of transporter trafficking including protein phosphatase A, Ca^++^, and tyrosine kinase-linked pathways. Recent findings on the dopamine and glutamate transporters demonstrated that PKC activation by PMA resulted in enhanced ubiquitination and endocytosis, while removal of ubiquitinated residues abolished PKC-dependent ubiquitination and endocytosis [11,12,13]. Although some proteins that interact with GlyT1 and GlyT2 have been reported, including syntaxin 1A, a member of the collapsing response mediator protein Ullip6, and syntenin-1, very little is known about their role in modulation of GlyTs trafficking [14,15,16].

Accumulating experimental evidence with closely-related transporters such as DAT, points to the *N*-terminal tail as the main region of protein kinase C-dependent posttranslational modifications such as phosphorylation and ubiquitination [12,17,18], suggesting a regulatory role of this *N*-terminal domain. By contrast for the glutamate transporter GLT-1, mutation of all 11 lysine residues within the amino and carboxyl-terminal domains abolished ubiquitination, showing a role for both termini in posttranslational modifications. Hence, in the present study we have analyzed whether PKC activation leads to GlyT1 ubiquitination. Surprisingly, PKC activation by PMA resulted in a time-dependent enhancement of ubiquitination and accelerated endocytosis. Site-directed mutagenesis studies revealed that simultaneous substitutions of all 6–7 lysine residues in the amino- and carboxy-terminus inhibited the PKC-dependent ubiquitination and endocytosis of three GlyT1 isoforms. Furthermore, removal of ubiquitination sites prevented GlyT1 transporter degradation but did not affect the PKC-dependent phosphorylation. Altogether, these data demonstrate redundancy in the mechanism of ubiquitination and suggest that PKC-dependent phosphorylation and ubiquitination may regulate different GlyT1 transporter properties.

## Materials and Methods

### Materials

[^3^H]-Glycine was purchased from Perkin Elmer Life Scientific (Boston, MA). Phorbol 12-myristate 13-acetate (PMA), *N*-ethylmaleimide, anti-FLAG M2 affinity gel and rabbit anti α-actin antibody were purchased from Sigma (St. Louis MO). Monoclonal anti-DAT antibodies were from EMD Millipore (Temecula, CA.). Ni-NTA agarose was from Qiagen (Hilden, Germany). Monoclonal mouse P4D1 to ubiquitin was from Santa Cruz Biotechnology (Santa Cruz, CA). Monoclonal antibody to EEA1 was purchased from BD transduction laboratories (San Jose, CA). Fluorescently or HRP-labeled secondary antibodies were from Jackson Immunoresearch Laboratories, Inc. (West Grove, PA). Rabbit polyclonal antibodies to GlyT1 were kindly donated by Detlev Boison and Dietmar Benke (University of Zurich).

### Plasmid constructs and mutations

The cDNA encoding for the mouse glycine transporter (mGlyT1) was purchased from the American Type Culture Collection (ATCC) and the human GlyT1b and GlyT1c cDNAs were kindly provided by Professor Bruno Giros (INSERM, France). The gene encoding for the human dopamine transporter (DAT) was provided by Dr. A. Sorkin (University of Pittsburg). All GlyT1 isoforms were tagged with Flag and 10X-His (FH) epitopes at the *N*-termini and the resulting constructs cloned into pCDNA 3.1 (FH-GlyT1) as previously described [11]. Single and multiple lysine substitutions were made using the FH-GlyT1 or FH-DAT as templates and a QuickChange site-directed mutagenesis kit following the manufacturer’s protocol (Stratagene Cloning Systems, La Jolla, CA) and the mutations were verified by automatic dideoxynucleotide sequencing.

### Cell culture and transfections

Porcine aortic endothelial (PAE-C, available from ATCC) cells were kindly provided by Dr. A. Sorkin (University of Pittsburg) and grown at 37°C and 5% CO_2_ in Ham’s F12 medium containing 10% FBS and antibiotics (Life Technologies, Grand Island, NY). PAE cells were grown to 50–80% confluence and transfected with appropriate plasmids using Effectene according to the manufacturer’s recommendation (Qiagen, Hilden, Germany). PAE cells stably expressing wild-type or GlyT1 mutants were selected by growing them in the presence of G418 (400 μg/ml).

### Affinity chromatography and western blotting

Affinity chromatography was performed as previously described [11]. Briefly, PAE cells stably expressing WT or mutant FH-GlyT1 were grown for two days in 35 mm dishes to 100% confluence and treated with vehicle (DMSO) or PMA for different periods of time. The cells were placed on ice for 5–10 min and washed three times with Ca^2+^- and Mg^2+^-free cold phosphate-buffered saline (PBS), and the proteins solubilized in lysis buffer (1% Triton X-100, 25 mM HEPES, pH 7.6, 10% glycerol, 100 mM NaCl, 10 mM sodium fluoride, 1 mM phenylmethylsulfonyl fluoride, 10 μg/ml leupeptin, 10 μg/ml aprotinin, 10 mM *N*-ethylmaleimide, 15 mM imidazole) for 10 min at 4°C. The cleared lysate was incubated with Ni-NTA for 1 h, the beads washed five times with lysis buffer and FH-GlyT1 eluted with 300 mM imidazole in lysis buffer. The eluate was diluted 10 times with the FLAG binding buffer (50 mM Tris, 150 mM NaCl, 10% glycerol, 1% Triton) and incubated for 4 h with FLAG M2 affinity gel. The mixture was washed five times with 1 ml of FLAG binding buffer, and GlyT1 was eluted with 0.1 M glycine (pH 2.8). The eluted fraction was quickly mixed with loading dye and boiled for 5 min. Total lysates or purified GlyT1 were subjected to electrophoresis in 8.0% SDS-polyacrylamide gels, and the proteins transferred to nitrocellulose membrane. Membranes were blotted with monoclonal mouse antibodies against ubiquitin and rabbit antibodies against GlyT1, followed by corresponding secondary antibodies conjugated with horseradish peroxidase, and detection using the enhanced chemiluminescence kit from Pierce (Thermo Fisher Scientific, Rockford, IL). Quantification was performed using densitometry and Adobe Photoshop software.

### Retina preparation and primary cultures

Adult and newborn (Postnatal day 1–5, P1-5) of C57BL/6J black mouse were used for histology and preparation of primary cultures, respectively. All animal use procedures were in conformance with the Guide for Care and Use of Laboratory Animals (National Institute of Health). These procedures were performed under a protocol approved by the University of Texas at El Paso (UTEP) Institutional Animal Care and Use Committee (IACUC). Adult animals were deeply anesthetized by intraperitoneal injection of pentobarbital (50 mg/kg) and the eyes enucleated and open by cutting along the ora serrata. Following removal of the vitreous, the posterior eyecup was fixed for 10 min by immersion in 4% paraformaldehyde in PBS. After fixation, the eyecups were cryoprotected by immersion in 10 and 20% sucrose followed by a final incubation of 30% sucrose in PBS overnight at 4°C. The following day, the optic cups were embedded in tissue-tek, and frozen at -20°C. Vertical sections (15 μm) were obtained using a cryostat and placed onto gelatin-coated slides. Retinal primary cultures were prepared as previously described [19]. Cells were cultured on coverslips coated with 10 μg/ml laminin and 0.01% poly-L-lysine and grown at 37°C and 5% CO_2_ in Neurobasal A medium supplemented with B27, 0.5 mM Glutamax and antibiotics (Life Technologies, Grand Island, NY). Neurons were allowed to differentiate and used after 10 days in culture.

### Immunofluorescence staining and microscopy

The PAE cells were grown on glass coverslips and treated with DMSO or 1 μM PMA for 30–60 min at 37°C. After treatment, the cells were washed with CMF-PBS, fixed with freshly prepared 4% paraformaldehyde for 15 min at room temperature and mildly permeabilized using a 3-min incubation in CMF-PBS containing 0.1% Triton X-100 and 0.5% bovine serum albumin at room temperature. The cells were then incubated in CMF-PBS containing 0.5% bovine serum albumin at room temperature for 1 h with primary antibodies, and subsequently incubated for 60 min with secondary antibodies labeled with CY3 or Alexa-488 (Jackson Laboratories, West-Glove, PA). Both primary and secondary antibody solutions were precleared by centrifugation at 100,000 *x g* for 20 min. After staining, the coverslips were mounted in Mowiol (Calbiochem). Images were acquired with a Zeiss inverted fluorescence microscope, equipped with an AxioCam MRm from Carl Zeiss, filter wheel and a Xenon 175 W light source, assisted with the Axiovision software 7.1 (Carl Zeiss, New York, NY). High resolution digital images were acquired through the corresponding filter channels (Alexa-488 and CY-3 filters), and the final arrangement of all images was performed using photoshop software. Images from tissue sections and transfected PAE cells were acquired with a Zeiss laser scanning confocal microscope (LSM700), assisted with the ZEN 2009 software (Carl Zeiss, New York, NY). Alexa-488 and Cy3 fluorophores were excited with 488 nm and 555 nm lasers respectively, and high resolution optical section images were acquired and processed for quantitation of co-localization for Alexa-488 and Cy3 fluorescence signals, on a pixel by pixel bases, from 15 endosomes in different cells, using the co-localization module of ZEN 2009 software [20].

### Surface Biotinylation

PAE cells expressing GlyT proteins were grown in 35-mm dishes and biotinylated as described previously [12,21]. Briefly, the cells were washed with cold PBS containing 0.1 mM CaCl2 and 1 mM MgCl2 (PBS) and incubated for 20 min on ice with 1.5 mg/ml sulfo-N hydroxysuccinimidobiotin (EZ-Link sulfo-NHS-biotin, Pierce) in PBS, followed by a second incubation with fresh sulfo-NHS-biotin. After biotinylation, the cells were washed twice with cold PBS, incubated on ice with 0.1 M glycine in PBS, and washed with PBS again. The cells were then solubilized in lysis buffer supplemented with 10 mM Tris-HCl (pH 7.5) at 4°C. The lysates were cleared by centrifugation for 10 min and the biotinylated proteins were precipitated with NeutrAvidin beads (Pierce), washed five times with lysis buffer (pH 8.0), and denatured by heating the beads in sample buffer at 95°C for 5 min. To precipitate non-biotinylated proteins, supernatants from the NeutrAvidin precipitation were further subjected to Ni-NTA affinity chromatography. The precipitates were washed five times with lysis buffer, the protein was then eluted in lysis buffer containing 300 mM imidazole and denatured by heating in sample buffer. The NeutrAvidin beads and Ni-NTA precipitates were subjected to SDS-PAGE and Western blotting with GlyT1 antibodies. Quantifications were performed using densitometry and Photoshop software.

### Glycine uptake assay

Glycine uptake was performed as previously described with the following modifications [22]. PAE cells were grown to 90–100% confluence and washed three times with HEPES buffer (10 mM HEPES pH 7.4, 135 mM NaCl, 2 mM KCl, 1 mM CaCl_2_, 1 mM MgSO_4_, and 10 mM glucose). Glycine uptake was initiated by the addition of 0.25 ml HEPES buffer containing 4 μCi of [^3^H]-glycine/ml and 400 μM cold glycine. After 10 minutes at 37°C, the buffer was removed and cells washed twice with ice-cold buffer following by extraction with 0.2 N of NaOH. Glycine uptake was determined by scintillation spectroscopy and specific glycine uptake is defined as the difference between total glycine uptake and minus glycine uptake measured simultaneously from parental cells transfected with pCDNA3.1. Protein concentration was determined as described by Bradford [23].

### Protein Degradation Assay

Wild type and GlyT1 mutants expressed in PAE cells were grown to 90–100% confluency. The cells were incubated for 2 h with cycloheximide (50 μg/ml) to stop the delivery and synthesis of new proteins followed by treatment with DMSO (6 h) or 1 μM PMA for 6, 4, or 2 h. After incubations, the cells were washed and lysed as previously described. The cleared lysate was quickly mixed with loading dye and the proteins denatured by heating as previously described [10,11,21]. It is worth mentioning that glycine transporters expressed in cultured cells is not aggregated by heating, unlike the endogenous from tissue that aggregates. Total lysates were subjected to electrophoresis and western blotting with rabbit antibodies to GlyT1 and α-actin, followed by corresponding secondary antibodies conjugated with horseradish peroxidase. Protein detection was performed with the enhanced chemiluminescence kit from Pierce (Thermo Fisher Scientific, Rockford, IL). Quantification was performed using densitometry and Adobe Photoshop software.

## Results

### GlyT1 endocytosis and ubiquitination

Several studies have demonstrated the role of PKC on endocytosis and posttranslational modifications of most transporters of the SLC6 family including GlyT1 [10,24,25]. For the dopamine transporter, we previously demonstrated that accelerated endocytosis triggered by PKC activation was a result of enhanced DAT ubiquitination [12,25], events further confirmed by other groups [26]. In this study, we analyzed PKC-dependent GlyT1 ubiquitination and endocytosis in three *N*-terminal isoforms (mouse GlyT1a and human GlyT1b and c isoforms) that differ in their length and in the number of lysine residues present in this region (Fig 1A).

The selected isoforms were analyzed in stably transfected PAE cells, allowing a detailed analysis of GlyT1 postranslational modifications in a highly homogenous cell population, where the majority of GlyT1 transporter is located at the plasma membrane and minor amounts are found in the endoplasmic reticulum. In our previous study, we reported that tagging the *N*-terminus of GlyT1a and GlyT1b with Flag and His did not affect the functional and trafficking properties of the transporter [10]. As shown in Fig 1B, FH-GlyT1b appear at the plasma membrane in vehicle-treated cells and accumulated into early endosomes labeled by EEA1 after incubation of the cells with 1 μM PMA at 37°C for 30–60 min, demonstrating that *N*-terminal tagging of GlyT1 did not affect retrograde and anterograde trafficking (Fig 1B [10]). Similarly, FHGlyT1c, included in this study, localized mainly to the plasma membrane in non-stimulated cells and minor co-localization with EEA1 was found in early endosomes. As with GlyT1b, PKC-dependent GlyT1c endocytosis was observed after incubation of the cells with PMA for 30–60 min, with GlyT1 accumulated in a large number of EEA1-positive early endosomes, as observed by co-localization (Fig 1C, see insets). This event was blocked by pre-incubation of the cells with 1 μM of the PKC specific inhibitor BIM for 30 min, demonstrating the PKC-dependence, as demonstrated in previous studies [10,12]. After quantification of the co-localization signal, we found a significant increase in co-localization after stimulation of PKC, as suggested by the increase in the Manders’ overlap coefficient and a 5–10 fold increase in fluorescence intensity (Fig 1D).

**Fig 2:**
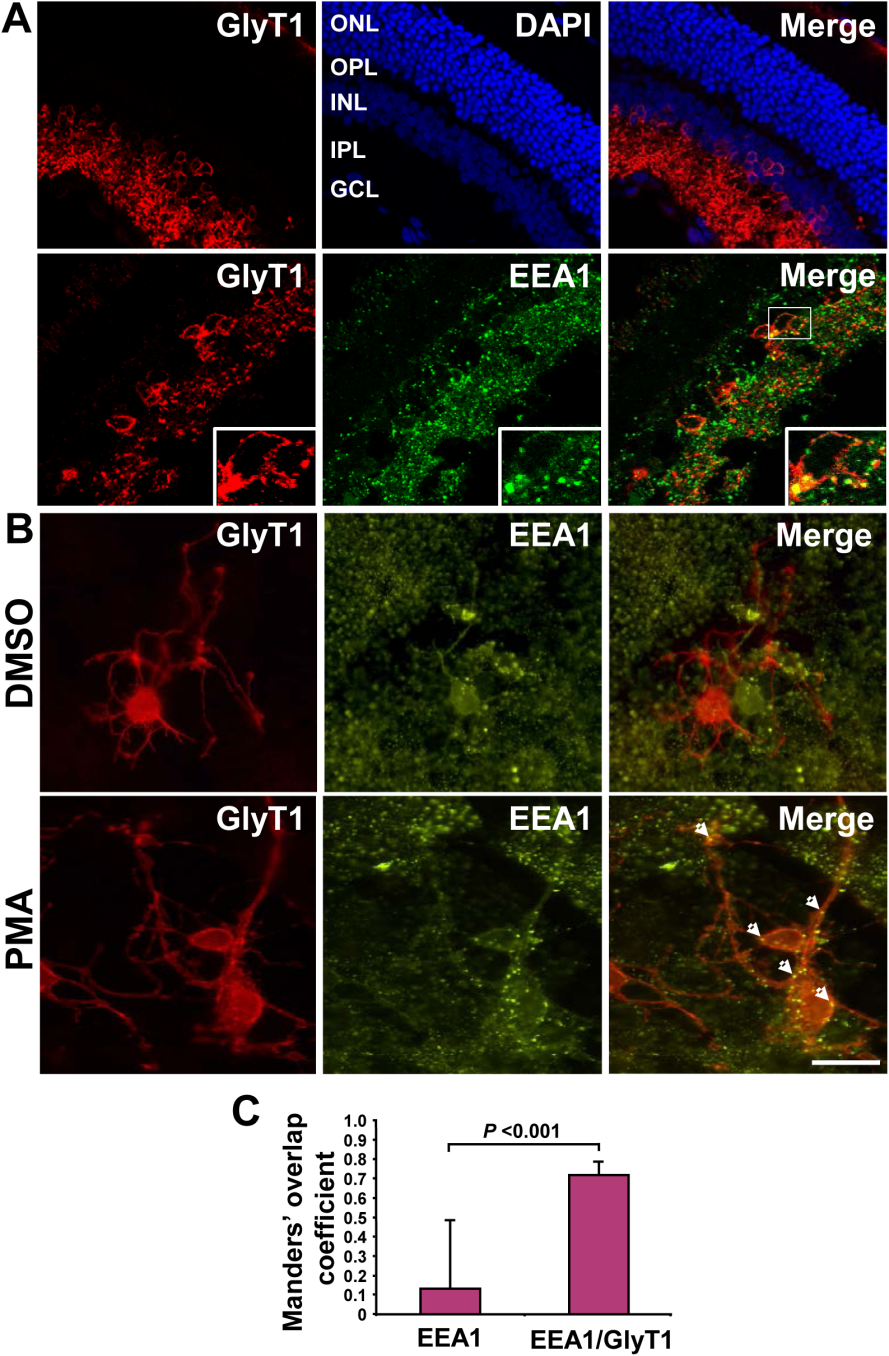
Localization of GlyT1 in mouse amacrine neurons. **A**) Vertical sections from adult C57BL/6J mouse retinas were stained for GlyT1, DAPI and EEA1, and analyzed by confocal microscopy. DAPI staining depicts the nuclei in cell bodies of the retina layers. Outer Nuclear Layer, ONL; Outer Plexiform Layer, OPL; Inner Nuclear Layer, INL; Inner Plexiform Layer, INL and Ganglion cell layer, GCL. **B)** Retinas from neonatal mouse were isolated, the tissue digested with papain and the cells plated on poly-L lysine and laminin- coated glass coverslips. Primary cultures were incubated with DMSO or 1 μM PMA for 1 h followed by detection of glycinergic amacrine neurons by immunostaining with GlyT1 and co-localization with EEA1. Single 0.65 μm optical sections were acquired by confocal microscopy and analyzed with ZEN 2009 software, as described in Fig 1D. *Scale bars*, 10 μm. **C)** Co-localization was measured for 10 EEA1- and 10 EEA1/GlyT1-positive endosomes from retinal sections depicted in panel A. Data is represented as described for Fig 1D and analyzed by Student’s *t*-test.

Several studies have described the regions in the mouse and rat central nervous systems that are enriched in glycinergic neurons such as the spinal cord, cerebellum and brain stem but the projections and connections are still not well defined. [6,27,28]. By contrast, the precise localization of glycinergic cell bodies, dendrites and connection are better characterized in the mammalian retina [29,30]. Therefore, to investigate whether endocytosis is a molecular event that takes place in glycinergic neurons, we used sections of mouse retina and primary cultures of mouse glycinergic amacrine neurons to study GlyT1 trafficking to early endosomes. To this extent, we obtained 15 μm retinal vertical sections and stained them with DAPI and antibodies specific for GlyT1 or EEA1. As shown in Fig 2A, three layers of cell bodies were clearly demarcated, corresponding to 1) the cell bodies of cones and bipolar cells (ONL, outer nuclear layer); 2) the middle layer accounting for cell bodies of horizontal and amacrine cells (INL, inner nuclear layer), and 3) the cell bodies of ganglion cells (GCL). Strong GlyT1 immunoreactivity was observed most prominently around cell bodies in the inner nuclear layer and descending dendrites to the inner plexiform layer that contact the ganglion cells. This arrangement clearly demonstrates the integrity of the sections and specific GlyT1 staining within the glycinergic amacrine neurons. As observed in Fig 2A, bottom panel, EEA1 staining appeared as puncta spread throughout all the retinal layers, which corresponded to early endosomes. Interestingly, a few discrete puncta showed co-localization between GlyT1 and EEA1 in 0.65 μm confocal optical sections and demonstrated by quantitative overlap coefficient analysis (Fig 2C), suggesting that GlyT1 uses early endosomes for endocytosis.

To get insights into the PKC-dependent endocytosis in neurons, we prepared primary cultures from P1-P3 mouse retinas and after 10 days in culture, the cells were incubated with vehicle DMSO or PMA for 1 h. As illustrated in Fig 2B, GlyT1 staining clearly defines the plasma membrane in cell bodies of amacrine neurons and appears in a few intracellular vesicles in DMSO treated cells. We observed a few vesicles inside the cell body that showed co-localization with EEA1, likely representing constitutive endocytosis, as has been described for the endogenous dopamine transporter [25,31]. By contrast, we could show a modest increased number of endosomes with co-localization between those proteins after PKC activation with PMA, mainly in cell bodies and a few in neuronal varicosities (see arrowheads in Fig 2B). However, a clear and significant effect in enhancement of endocytosis by PMA could not be determine in neurons given that, in non-stimulated cells, a large number of EEA1 positive endosomes contained low levels of GlyT1. Unfortunately, the low yields of GlyT1 protein recovered from the combination of several primary cultures were not sufficient to perform biochemical studies, likely due to the low amount of glycinergic neurons obtained from developing animals. Thus, preparation of primary cultures from brain regions enriched in GlyT1 expression could allow biochemical analysis; however, the precise location of glycinergic circuits is still under development. Nevertheless, these results suggest that GlyT1 endocytosis into EEA1 endosomes occurs in neurons from the retina and that PKC activation may increase the localization of GlyT1 in endosomes.

**Fig 3:**
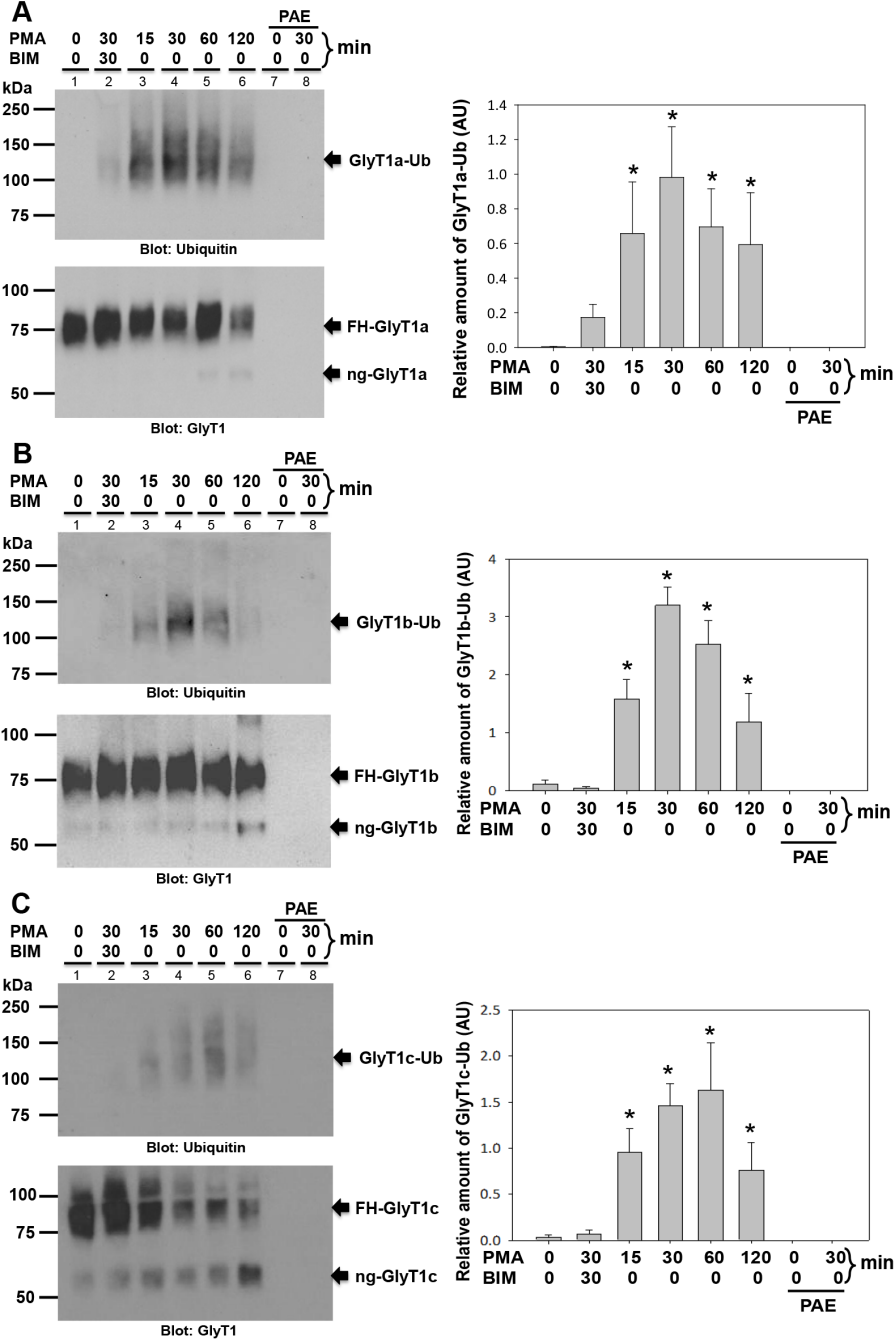
Time course of PKC-induced ubiquitination of GlyT1 isoforms. **A)** PAE cells stably expressing FH-GlyT1a were incubated with PMA (1μM) for 0 to 120 min. After incubation, the proteins were solubilized and GlyT1 purified by tandem affinity chromatography using Ni-NTA-agarose and FLAG M2 gel. Purified GlyT1 transporter was subjected to SDS-PAGE and western blotting using ubiquitin and GlyT1 antibodies. **B)** FH-GlyT1b isoform. **C)** FH-GlyT1c isoform. Blots were subjected to densitometry analysis using image J software and the relative amount of ubiquitinated GlyT1 was normalized to the total GlyT1 transporter. The Y axes represent the relative amount of ubiquitinated GlyT1. Data are expressed as the mean ± SEM, n = 4. ** A value of *p*<0.05 (*) was obtained when each experimental sample was compared with untreated control cells *via* one-way analysis of variance (ANOVA) and Student’s *t*-test. *GlyT1-Ub*, ubiquitinated glycine transporter; *FH-GlyT1*, Flag, His-tagged glycosylated glycine transporter; *ng-GlyT1*, non-glycosylated glycine transporter.

To investigate the role of ubiquitination on PKC-dependent endocytosis of the three *N*-terminal GlyT1 isoforms, we used wild type and mutants expressed in stable PAE cell lines. By using stable cell lines, we are sure to work with a homogenous cell population where the majority of the cells have similar levels of plasma membrane expression and guarantee consistency from experiment to experiment. To investigate whether GlyT1 isoforms were ubiquitinated in response to PKC, PAE cells expressing the FH-GlyT1s were incubated with PMA for various lengths of time and the transporter isoforms were purified to near homogeneity and analyzed by western blotting. As shown in Fig 3A–3C, when GlyT1 was purified from DMSO-treated cells and analyzed with ubiquitin-specific antibodies, a faint smear was detected at ~90–120 kDa, depending on the isoform. By contrast, when GlyT1 was purified from PMA-treated cells, a dramatic increase in ubiquitin immune-reactivity was observed for all isoforms (a, b and c) with maximum signal at 30 min, maintained at 60 min and decreased with continued incubation time, as depicted after normalization of ubiquitinated GlyT1 for the total GlyT1 transporter, in the densitometry analysis (Fig 3). In addition, enhanced GlyT1 ubiquitination was abolished by 30 min pre-incubation with the PKC inhibitor BIM prior to stimulation with PMA, demonstrating a PKC-dependent mechanism. Western blotting with GlyT1 antibodies confirmed the presence of FH-GlyT1 migrating as a smeared band at ~70–90 kDa, in agreement to that observed for tagged and untagged GlyT1 isoforms. The results of the densitometry analysis showed higher levels of ubiquitinated GlyT1b and GlyT1c compared to those for GlyT1a (represented as an increase in arbitrary units). This difference can be accounted by the extra lysine residues in the isoforms b and c, available for ubiquitination. These data together suggest that PKC activation triggers GlyT1 ubiquitination and endocytosis in a time dependent fashion.

### Mutations of lysine residues in the *N*- and *C*-termini abolish ubiquitination and endocytosis

Ubiquitination and phosphorylation of several transporters such as DAT, NET and SERT have been shown to involve residues at either *N*- or the *C*-terminus of these proteins. A recent study expressing the rat GlyT1b isoform in transiently transfected MDCK cells by Fernandez-Sanchez *et al*. [24], showed PKC-dependent ubiquitination and proposed that the *C*-terminal tail of the rat GlyT1b isoform contains endocytic determinants, pointing to Lys-619 as responsible for ubiquitination and internalization. By contrast, we and others have reported the presence of multiple lysines as ubiquitin-conjugation sites in the dopamine and glutamate transporters [12,13], pointing to multiple lysine residues as potential conjugation sites rather than a single lysine residue.

To identify the ubiquitin conjugation sites for the GlyT1 isoforms (Fig 1A), we performed site-directed mutagenesis followed by endocytosis and ubiquitination analysis. Fluorescence microscopy analysis showed that the single and pairwise lysine mutations did not impair the trafficking when they were expressed in PAE cells, since GlyT1 mutants were efficiently delivered to the plasma membrane, and not affected in endocytosis by PMA. Similarly, western blot analysis demonstrated that these mutants were not affected in PMA-induced GlyT1 ubiquitination, including mutations at the homologs residue to K619 (data not shown).

**Fig 4:**
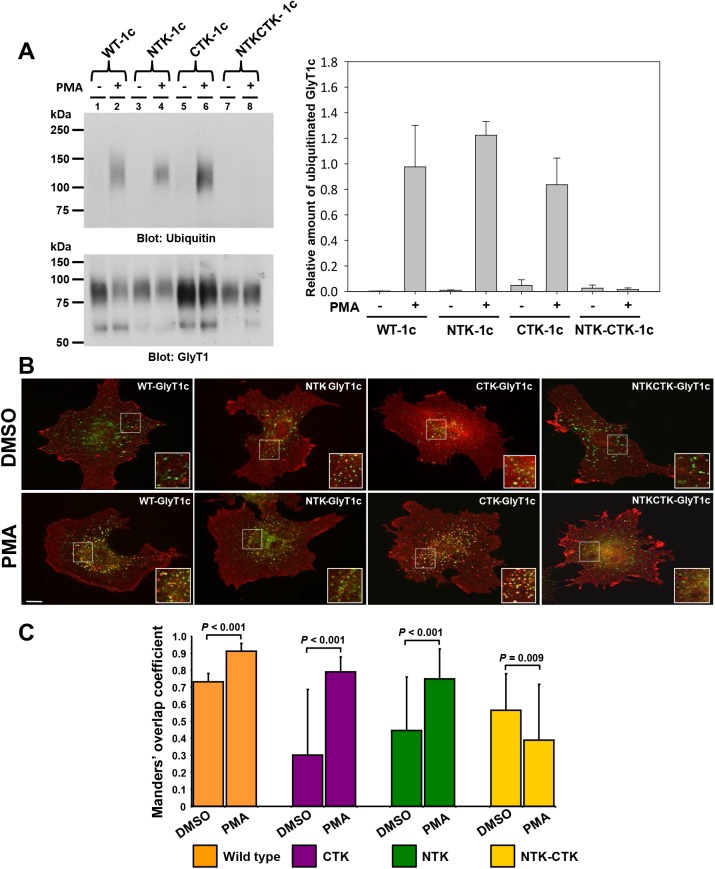
Ubiquitination and endocytosis of multi-lysine GlyT1c mutants. **A)** PAE cells expressing WT FH-GlyT1c, NTK-1c, CTK-1c, and NTK-CTK-1c were incubated with DMSO or PMA (1μM) for 30 min. After incubation, the proteins were solubilized and GlyT1 purified by tandem affinity chromatography using Ni-NTA-agarose and FLAG M2 gel. Purified transporter was subjected to SDS-PAGE and western blot with ubiquitin and GlyT1 antibodies. Densitometry analysis was performed as described for Fig 3 and expressed as a mean ± SEM, n = 4. **B)** PAE cells expressing WT FH-GlyT1c, NTK-1c, CTK-1c, and NTK-CTK-1c were incubated with DMSO or PMA (1 μM) for 30 min. at 37°C, fixed and immunostained with anti-GlyT1 and anti-EAA1 antibodies followed by incubation with a CY-3 and Alexa 488 labeled secondary antibodies, respectively. A z-stack of optical sections was acquired through YFP (green) and CY3 (red) filter channels. Single optical sections through the middle of the cells are shown. ‘Yellow’ in the merged images signifies co-localization of CY3 (GlyT1) and YFP (EEA1). Images were selected to represent the cell population. *Scale bars*, 10 μm. C) Manders’ overlap coefficient of merged images captured from doubly-labeled PAE cells with anti-GlyT1 (red) and anti-EEA1 (green) antibodies; a value of 1 represents 100% of both fluorescence signals co-localized in all the pixels involved in the regions of interest (ROIs). Values are presented for 15 randomly selected endosomes in different cells from wild-type and mutants. Statistical analysis was performed as described in Fig 1.

We therefore expected that several lysine residues were able to function as ubiquitin acceptor sites. For that reason, we stably expressed in PAE cells GlyT1 mutants devoid of any lysine residues in the amino and/or carboxy-terminal tails and analyzed the ubiquitination levels by western blotting. Although we present in Fig 4 the results obtained for GlyT1c isoform, it is worth mentioning that the same findings were obtained for the isoforms GlyT1a and b (data not shown). As shown in Fig 4A, even substitution of all of the lysine residues in either the amino (NTK) or carboxy (CTK) terminal tail with arginines failed to prevent ubiquitination for all GlyT1 isoforms. By contrast, substitutions at both tails (NTK-CTK) abolished the PKC-dependent ubiquitination to levels similar to those obtained for GlyT1 purified from non-stimulated cells. Interestingly, the mouse GlyT1a *N*-terminus contains two lysines that are consecutive (K20 and K21) and two that are closely spaced (K4 and K7). Combination of single or pairwise substitutions at these residues, in a mutant devoid of lysine residues at the *C*-terminus, also failed to abolish ubiquitination (data not shown), discarding a possible role of two closely spaced lysines as responsible for ubiquitination, as suggested previously [12]. This is consistent with findings described for the dopamine transporter, in which pairwise mutations of ubiquitin acceptor sites (K19, K27 and K35) did not abolish ubiquitination of DAT [12]. These results clearly demonstrate that any lysine residues present in either tail represent potential sites to conjugate with ubiquitin moieties and may serve as an acceptor site.

Consistent with these findings, fluorescence microscopy analysis of the NTK and CTK mutants showed that the mutant transporters trafficked to the plasma membrane and endocytosed into early endosomes in response to PMA stimulation, at rates similar to the wild-type GlyT1 (Fig 4B). In contrast, removal of all *N*- and *C*-terminal lysine residues in the NTK-CTK mutant abolished the PMA-induced endocytosis of the transporter and co-localization between transporter and EEA1 was no longer observed. These results together suggest not only that endocytosis of GlyT1 is dependent on ubiquitination but also that this post-translational modification is redundant and involves conjugation of ubiquitin to any of the multiple potential conjugation sites at, either the *N*- or *C*-terminal tails, rather than to a specific lysine residue. Interestingly, similar findings were described for the plasma membrane glutamate transporter, where endocytosis was only abolished after 11 lysine substitutions from both *N*- and *C*-terminal tails [13].

### Internalization and degradation of GlyT1 is impaired by lysine substitutions

**Fig 5:**
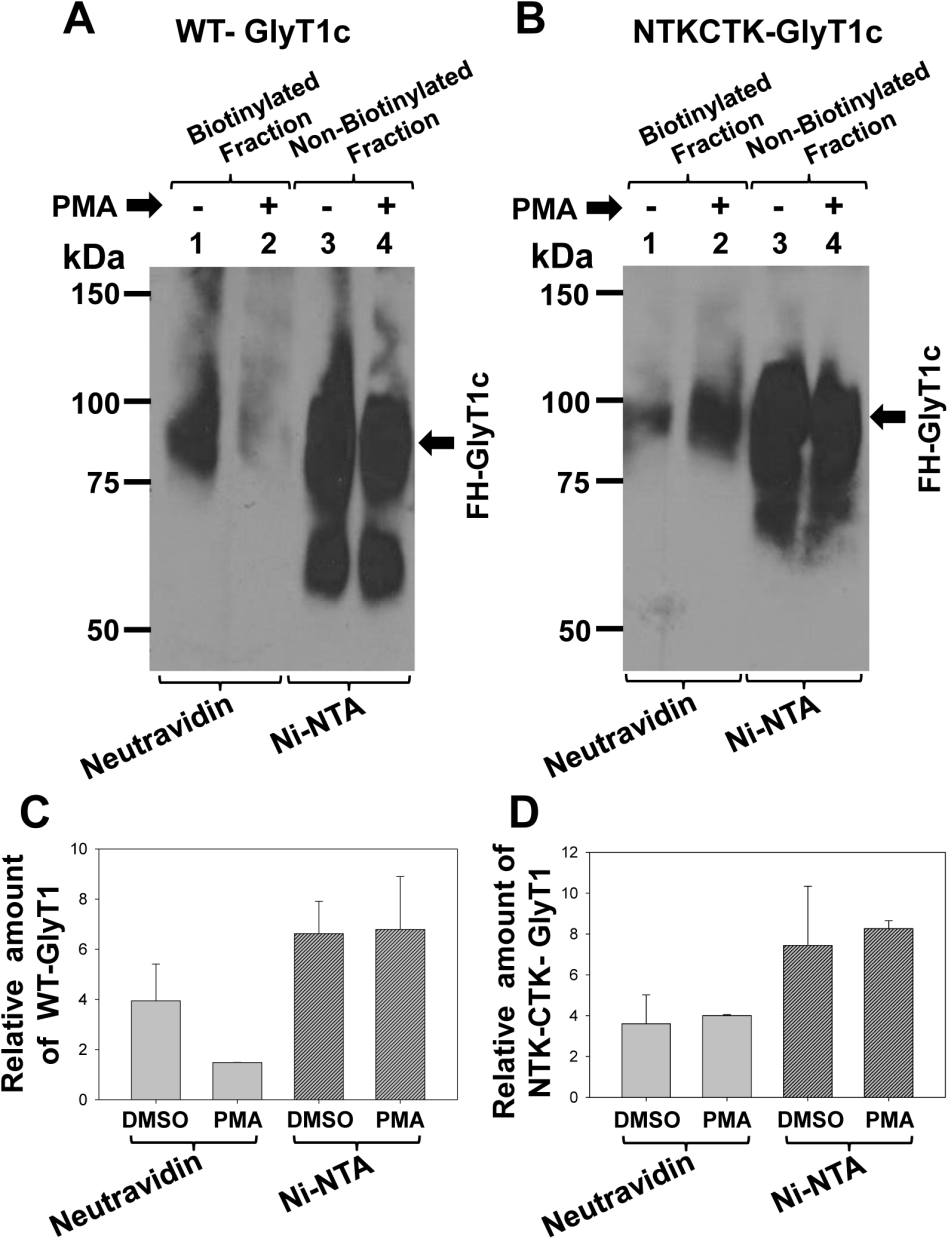
Multi-lysine mutations affect the levels of cell-surface GlyT1. PAE cells expressing **A)** WT or **B)** NTKCTK-GlyT1 were incubated with DMSO or PMA (1 μM) for 30 min at 37°C. The cells were subjected to cell surface biotinylation, and biotinylated proteins were pulled down with Neutravidin (NeuAv) beads. Non-biotinylated proteins were purified from NeuAv supernatants using Ni-NTA agarose. NeuAv and Ni-NTA precipitates were separated on SDS-PAGE, transferred to nitrocellulose and the blots were probed with GlyT1 antibodies. **C)** Quantification of the amount of biotinylated and non-biotinylated GlyT1. The densitometry analysis was performed using ImageJ and the values are expressed as the mean ± SEM, n = 2.

To determine whether mutations at ubiquitination sites prevented endocytosis and degradation of GlyT1, we incubated cells expressing the WT GlyT1c isoform or the mutant NTK-CTK with vehicle DMSO or PMA for 30 min. We then labeled the total cell surface proteins by incubation of the cells with the impermeant reagent sulfo-N-hydroxysuccinimido biotin. Total biotinylated cell surface proteins were precipitated with Neutravidin beads and biotinylated proteins subjected to western blotting with GlyT1-specific antibodies. As shown in Fig 5A, PMA induced a consistent 50–70% reduction in WT transporter at the cell surface when compared to vehicle-treated cells. In the other hand, PMA treatment of cells expressing the NTK-CTK mutant showed no change in the amount of cell surface transporter, as compared to non-treated cells, suggesting that ubiquitination is critical for accelerated endocytosis (Fig 5B). This data is consistent with a poor localization of the mutant transporter NTKCTK into early endosomes in PMA-stimulated cells (Fig 4B).

**Fig 6:**
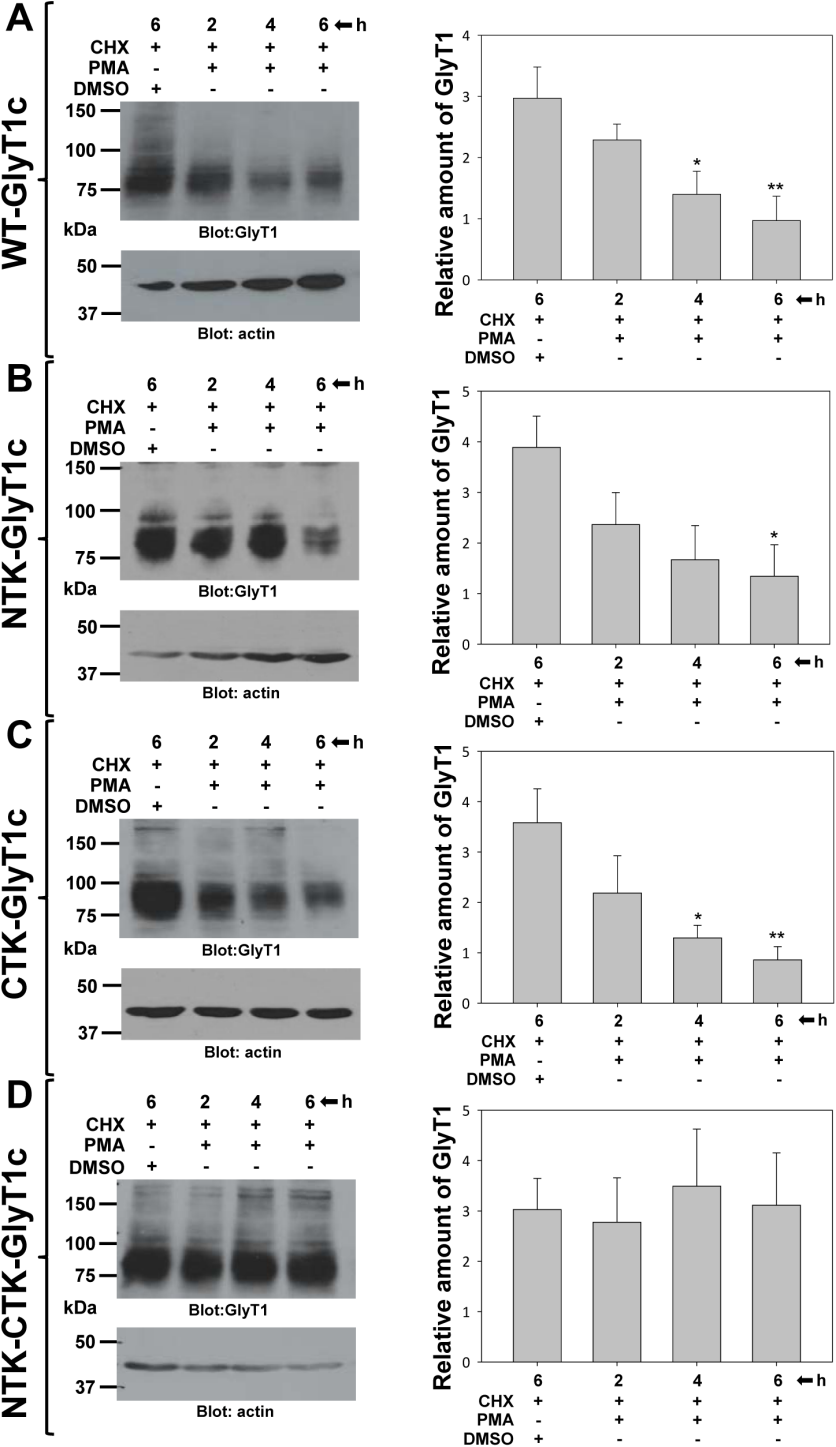
PKC-dependent GlyT1 degradation is impaired by lysine mutations. PAE cells expressing WT and mutant forms of GlyT1 were incubated with 50 μg/ml of cycloheximide (CHX) for 2 h followed by treatment with 1μM PMA for 0–6 h. In all conditions, cells were incubated in the presence of CHX for a total of 8 h. After incubations, the cells were lysed and total lysates subjected to SDS-PAGE and western blot with GlyT1 and actin antibodies. **A)** WT-FH-GlyT1c, and mutants **B)** NTK-1c, **C)** CTK-1c, and **D)** NTK-CTK-1c. Densitometry was as described in Fig 3 and values expressed as a mean of three to five independent experiments ± SEM, n = 3–5. A value of *p*<0.05 (*) was obtained from each experimental sample, as compared with untreated control cells, *via* one-way analysis of variance (ANOVA) and Student’s *t*-test.

It has been demonstrated previously that PKC activation triggers endocytosis and degradation of the dopamine and glutamate transporters and that removal of ubiquitin-conjugating sites abolished these processes [12,13,32]. To test whether PKC-dependent ubiquitination and endocytosis result in GlyT1 degradation, we incubated WT GlyT1c or mutants NTK, CTK or NTK-CTK with cycloheximide (CHX) for two hours to abolish new protein synthesis and allow the delivery of previously synthesized transporter to the plasma membrane, followed by the addition of PMA for 2, 4 or 6 h or DMSO for 6 h. In these experiments, at the end of the incubation time with PMA or DMSO, all of the cells had been treated with CHX for a total of 8 h (Fig 6A–6D). The cells were lysed and proteins analyzed by SDS-PAGE and western blotting with GlyT1 antibodies. As illustrated in Fig 6A (lanes 1–4), the WT GlyT1c was degraded over time, with a half-life of ~4 h. Similar to the WT, PKC activation led to degradation of both NTK and CTK mutants, suggesting that any lysine residues present in either the *N*- or *C*-terminal tail can be ubiquitinated, thereby targeting the transporter for degradation (Fig 6B and 6C, lanes 1–4). Noteworthy, whereas NTK mutant showed a similar rate of degradation to the wild type, the CTK mutant had a diminished half-life of ~2 h. By contrast, similar levels of total transporter were detected after 2, 4, and 6 h incubation with PMA for the double mutant NTK-CTK, indicating that the loss of all possible ubiquitination sites resulted in stabilization of the transporter and suggesting that ubiquitination is the signal that targets GlyT1 transporter for endocytosis and further degradation (Fig 6D, lanes 1–4).

To rule out the contribution of altered turnover or any other potential off-effect of CHX treatment, we performed several control experiments in which we incubated WT GlyT1c or the lysine mutants with DMSO or PMA for 6 h in the absence of CHX. As shown in S1A–S1D Fig (lanes 1 and 2), cells incubated with vehicle DMSO showed a modest increase in the amount of transporter compared to those cells incubated with CHX, likely due to constant input of newly synthesized GlyT1 from the ER. In the other hand, incubation with PMA led a modest, non-significant, reduction in the total transporter compared to DMSO-treated cells. Although PMA is accelerating endocytosis and degradation in the absence of CHX, we cannot detect this degradation because it is covered by a strikingly enhanced delivery of GlyT1 from the ER to the plasma membrane. This enhancement in protein synthesis is highlighted by the increased accumulation of newly synthesized, non-glycosylated GlyT1 that migrated at 55–60 kDa (S1A, S1C and S1D Fig), which was present at very low levels, or absent (S1 Fig, panel B), in cells incubated with DMSO, and absent in the cells incubated with CHX. These results together demonstrate that PKC-dependent ubiquitination is the trigger for endocytosis and further degradation, and that the process of ubiquitination can take place at any lysine residue lying at either *N*- or *C*-terminus of the transporter.

### GlyT1 phosphorylation takes place independent of ubiquitination

**Fig 7:**
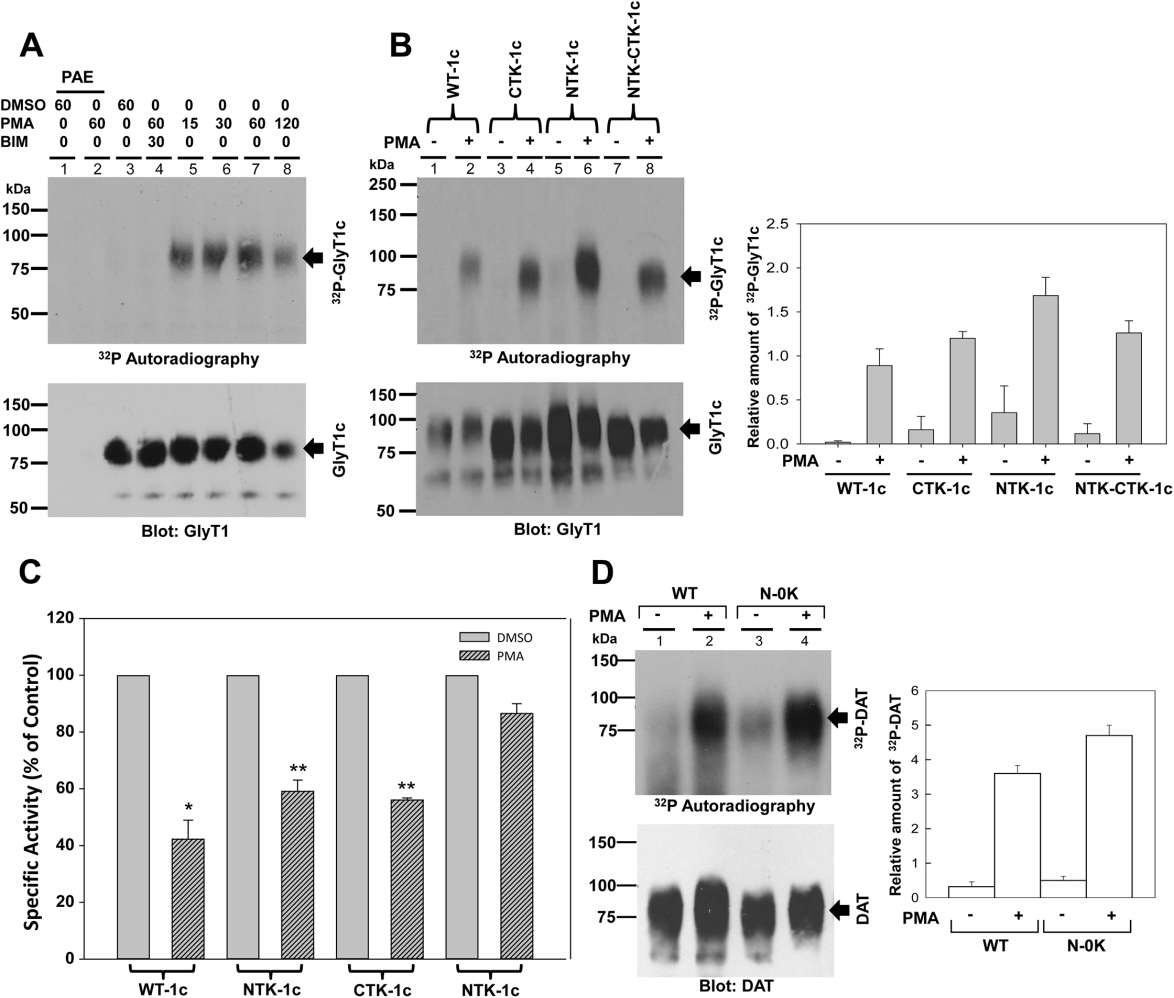
GlyT1 phosphorylation and glycine uptake. **A)** PAE cells stably expressing FH-GlyT1 were labeled with 50 μCi ^32^P-orthophosphate/ml followed by incubation with DMSO or 1 μM PMA for 0 to 120 min. Labeled GlyT1 was purified by tandem affinity chromatography and analyzed by autoradiography and Western blotting with GlyT1 antibodies. **B)** PAE cells expressing WT FH-GlyT1c, or the mutants NTK-1c, CTK-1c, and NTK-CTK-1c were labeled with 50 μCi ^32^P-orthophosphate/ml followed by incubation with DMSO or 1 μM PMA for 60 min and treated as described in A. The autoradiography and GlyT1 blots were subjected to densitometry analysis and the resulting values are expressed as mean ± SEM, n = 3, **C)** For uptake experiments, cells were incubated with vehicle (DMSO) or 1μM PMA for 30 min followed by a 10 min incubation with 400 μM of [^3^H]-Gly at 37°C. Values are represented as % of control DMSO for each cell line, calculated from the following average specific activities in nmol/min/mg of protein: WT-1c, 41.3+/-3; NTK-1c,51.2+/-6; CTK-1c 39.4+/-3: NTK-CTK-1c, 56.8+/- 4;. Error bars represent the mean ± SE, *n* = 3, *p = *0*.*002*, **p <0.001. **D)** PAE cells expressing WT-DAT, and the mutant DAT were labeled with 50 μCi ^32^P-orthophosphate/ml followed by incubation with DMSO or 1 μM PMA for 60 min. Total DAT was purified by tandem affinity chromatography and analyzed by autoradiography and Western blotting with DAT antibodies. Values are expressed as mean + SEM, n = 3. A value of *p*<0.05 was obtained when each experimental sample was compared with untreated control cells *via* one-way analysis of variance (ANOVA) and Student’s *t*-test.

We previously demonstrated that GlyT1 is phosphorylated in response to PKCβ activation along with a reduction in glycine uptake capacity. This reduction in uptake is likely due to endocytosis, as described below. To analyze the relationship between phosphorylation, ubiquitination and further reduction in glycine uptake, we next studied the effects of lysine substitutions on GlyT1c phosphorylation and uptake to define the relationship between phosphorylation, ubiquitination, and glycine uptake. In control experiments, we subjected PAE cells transfected with the vector or expressing the WT GlyT1c to ^32^P-ATP metabolic labeling, followed by DMSO or PMA stimulation. After incubation, the cells were lysed, and the transporter purified and subjected to SDS-PAGE and autoradiography. Consistent with our previous published findings, incubation of PAE cells transfected with pCDNA3.1 vector with DMSO or PMA for 60 min failed to identify in any radiolabeled band or immunoreactivitiy to GlyT1 antibodies (Fig 7A, lanes 1 and 2). In the other hand, a faint smear could be detected when GlyT1 was purified from DMSO-treated cells. By contrast, in PAE cells expressing WT GlyT1c stimulated with PMA for varying periods of time (15–120 min), we observed a dramatic increase in GlyT1 phosphorylation that decayed after 2 h (lanes 5–8). When cells were treated with BIM to inhibit PKC and stimulated with PMA, this increase in phosphorylation of WT GlyT1was lost (Fig 7A, lane 4), demonstrating again its PKC dependence.

GlyT1 phosphorylation and ubiquitination are posttranslational modifications dramatically enhanced by PKC activation. Whether they regulate different properties of transporter or both depend of each other is something that needs to be explored. To get insights into the role of ubiquitination on phosphorylation, we then used the same experimental approach to compare phosphorylation in PAE cells expressing the NTK, CTK, and NTK-CTK forms of GlyT1c. Cells were subjected to ^32^P-ATP metabolic labeling followed by PMA stimulation for 60 min as above. As depicted in Fig 7B, PMA stimulation led to a significant ~10 fold increase in WT GlyT1 phosphorylation compared to vehicle-treated cells, as determined by densitometry of three independent experiments (p = <0.05). Interestingly, the PKC-dependent increase in phosphorylation was not affected for the three mutants tested NTK, CTK and NTK-CTK (Fig 7B, lanes 4, 6, and 8), demonstrating that phosphorylation can take place independent of ubiquitination (Fig 7B). As depicted in Fig 7B and confirmed by the densitometry shown at right, a slight increase in basal and PKC-dependent phosphorylation was observed for the mutant NTK, which could be due to increased accessibility of phosphorylated residues. In any case, consistent with the WT, this mutant also exhibited an 8–10 fold enhancement of phosphorylation after PKC activation (Fig 7B, lane 6; p = <0.002). These results confirmed the ability of the mutant NTK-CTK of GlyT1 to be phosphorylated despite the lysine mutations and abolishment of ubiquitination, further underlining the separation between the phosphorylation and ubiquitination mechanisms under consideration here.

It has been previously postulated that transporter endocytosis could explain the reduction in glycine uptake observed after PKC activation. To examine whether glycine uptake was affected, we measured uptake of radiolabeled glycine in PAE cells expressing the WT and lysine mutant forms of GlyT1c following 30 min incubation of the cells with DMSO or PMA. As expected for the WT GlyT1, glycine uptake was reduced to ~40–50% in PMA-treated cells when compared to DMSO, while the NTK and CTK mutants showed a ~50–60% reduction in uptake, consistent with the ability of these mutants to undergo PKC-dependent endocytosis and further down-regulation of transporter activity (Fig 7C). By contrast, the mutant NTK-CTK showed only a modest 10% reduction in uptake after PMA stimulation that was not statistically significant, demonstrating that the reduction in uptake capacity is due to ubiquitin-dependent transporter endocytosis, as previously suggested. These results together clearly provide the first evidence that PKC-dependent phosphorylation takes place independent of ubiquitination and that these posttranslational modifications likely regulate different transporter properties. To lend support to this hypothesis, we subjected PAE cells expressing the dopamine transporter, either the WT DAT or a DAT mutant devoid of lysines at its *N*-terminus to ^32^P-metabolic labeling after PKC activation by 60 min incubation with PMA. This DAT mutant was previously shown to exhibit defects in PKC-dependent endocytosis and ubiquitination. As shown in Fig 7D, PMA treatment resulted in enhanced phosphorylation of both WT and the mutant DAT, suggesting that PKC-dependent phosphorylation of DAT occurs independent of ubiquitination and confirming that these separate PKC-dependent modifications are shared by several SLC6 family members, perhaps highlighting similar regulatory mechanisms between members of the family. In agreement with this hypothesis, our previous published data demonstrate that pharmacological inhibition of PKCβ abolished phosphorylation but had no effect on the PKC-dependent reduction of glycine uptake, suggesting that abolishment of transporter phosphorylation is not required for endocytosis [10]. Perhaps transporter ubiquitination involves activation of PKCα.

## Discussion

The mechanism of PKC-dependent endocytosis of neurotransmitters transporters has been intensively studied for more than two decades and it is becoming accepted that PKC activation triggers several changes in these carriers. Of these, the best studied modifications are phosphorylation and ubiquitination [12,13,25,33,34]. Interestingly, PKC-dependent ubiquitination of neurotransmitter transporters is emerging as the main mechanism to explain transporter endocytosis; and in many instances, it also explains transporter degradation.

Although we have previously shown for the dopamine transporter that three lysine residues at the *N*-terminus of the dopamine transporter serve as the main ubiquitin-conjugation sites, a specific consensus sequence that may serve as signal for ubiquitination is far from being described [12]. Likewise, previous studies on glycine transporters have shown that pharmacological activation of PKC trigger transporter endocytosis [22]. Based on this evidence regarding glycine transporters endocytosis, we analyzed the role of ubiquitination on endocytosis and mapped the location of ubiquitination sites in stably transfected cells using three different *N*-terminal splice variants of GlyT1. In agreement with results obtained for DAT and other transporters, we showed that GlyT1 ubiquitination is a redundant mechanism and that there is no preference for a specific lysine residue in the terminal tails of this transporter. We report that replacement of three conserved lysine residues at the *C*-terminal tail of GlyT1 did not affect PKC-dependent ubiquitination and endocytosis, nor did the replacement of those in the *N*-terminal tail. However, replacement of both *N*- and *C*-terminal lysines resulted in abrogation of PKC-dependent ubiquitination. These findings demonstrate that any available and exposed lysine in either the *N*- or *C*-terminal tail can serve as an ubiquitin acceptor site and only replacement of lysines at both *N* and *C*-termini abolish ubiquitination and endocytosis. It is worth mentioning that this redundancy in the mechanism of GlyT1 ubiquitination was observed for three isoforms, mouse GlyT1a, human GlyT1b, and human GlyT1c; although we present only the GlyT1c data here, this redundancy was independent on the number of lysines contained within the *N*- or C-terminal tails of each. The redundancy in the mechanism of lysine selection for ubiquitination has been also observed for the glutamate transporter GLT-1, where PKC-dependent ubiquitination was abolished only after substitution of 11 lysine residues in the *N*- and *C*-terminal tails with arginines [13].

Although these transporters DAT, GLT-11 and GlyT1 contain several potential ubiquitin acceptor sites, we believe that only one lysine per transporter molecule becomes K63 poly-ubiquitinated and this modification involves the attachment of a single chain of 2–3 ubiquitin moieties. The experimental evidence comes from our previous work on DAT, where we demonstrate by mass spectrometry analysis that the transporter undergoes K63 poly-ubiquitination. Moreover, the shift in molecular mass between the ubiquitinated and unmodified GlyT1 that is observed by western blot analysis corresponds to a consistent difference of 20–30 kDa, suggesting that two or three ubiquitin moieties are added to a lysine residue at the *N*- or *C*-terminus of GlyT1. Identical shifts in electrophoretic mobility have been reported for GLT-1 and DAT [11,13]. Also, our results show that removal of either the three lysines near the *C*-terminus on all GlyT1 isoforms or those near the *N*-terminus does not affect the increase in 20–30 kDa mobility of the ubiquitinated mutant GlyT1, again indicating that a single lysine residue is modified with 2–3 ubiquitin moieties.

Similar findings were observed for GLT-1, where the re-introduction of a single lysine, in a mutant devoid of lysine residues at the *N*- and *C*-terminus, was sufficient to induce the PKC-dependent endocytosis and ubiquitination. Not surprisingly, the shift in mobility of the ubiquitinated mutant form was identical to that of the WT GLT-1. These results together suggest a common mechanism for the PKC-mediated ubiquitination whereby a single accessible lysine residue is modified with 2–3 ubiquitin moieties followed by endocytosis into endosomes. The E3 ubiquitin ligase Nedd4-2 has been shown to be involved in neurotransmitter transporter ubiquitination, although it remains to be explored whether the E3 ubiquitin ligase recognizes a target lysine randomly or shows a preference for a specific lysine residue, for example the most exposed or accessible residue [25,35].

In spite of this redundancy in residues that can be ubiquitinated, it is important to note that transporter ubiquitination has been reported to take place at lysines located at or near either the *N*- or *C*-terminus of several neurotransmitter transporters including DAT, GLT-1, GlyT1b and GlyT2, pointing to these terminal tails as potential regulatory domains of various transporter properties, including trafficking [12,13,36]. Although lysine residues exist in the intracellular loops of neurotransmitter transporters, no evidence has been provided to suggest ubiquitination in those regions of the carriers. In addition, it is not surprising that PKC-dependent phosphorylation sites have been extensively identified, in or near either *N*- and/or *C*-terminal tails of several neurotransmitter transporters such as those for dopamine, glutamate, glycine and glutamine [17,18,35,37,38,39,40]. To date, accumulated experimental evidence demonstrates that PKC-dependent transporter phosphorylation is mediated by serine and threonine rather tyrosine phosphorylation [10,35]. The role of these modifications, mostly obtained for DAT, are related to changes in overall conformation and kinetic constants; for example, it has been suggested that phosphorylation of Ser-7 of DAT shifts the equilibrium from a high to a low affinity cocaine binding state whereas phosphorylation of Thr-53 is involved in the AMPH-mediated substrate efflux and modulation of *Vmax* [33,34]. It has also been suggested that acidic substitutions at Lys-422, Thr-419 and Ser-420 in the intracellular loop 2 of the GlyT2 abolishes the PMA-induced internalization; however, it has not yet been determined whether these residues undergo posttranslational modifications or these phenotypic effects are due to a change in transporter conformation nor how these mutations abolishes endocytosis [41]. By contrast, our results suggest that transporter ubiquitination is the main mechanism of endocytosis and that PKC-dependent phosphorylation is independent of ubiqutination and endocytosis, as has been suggested for DAT [10,18]. Altogether, these findings support our hypothesis that the transporter tails represent domains that are involved in regulation of transporter properties, highlighting ubiquitination as responsible for endocytosis and degradation whereas phosphorylation could be a mechanism to regulate substrate binding and translocation or the kinetic properties of these transporters.

In contrast to our results that suggest the presence of several ubiquitin acceptor sites in the tails of GlyT1 isoforms and the redundancy in transporter ubiquitination, a previous report on the rat GlyT1b isoform suggests that ubiquitination of K619 near the *C*-terminus played a prominent role in both constitutive and PMA-induced endocytosis. In that work, substitution of the three lysine residues in the *C*-terminus to arginine abolished both constitutive and PMA-induced endocytosis; surprisingly, identical results were obtained for the single mutant K619R, pointing to a single residue as responsible for GlyT1b ubiquitination and endocytosis [24]. Moreover, the same group reported a single lysine K791 ubiquitination in the *C*-terminal tail of GlyT2 as the main mechanism underlying the PKC-induced endocytosis. These data suggest that GlyT1 and GlyT2 transporter ubiquitination is specific for a single lysine residue at the *C*-terminus [40]. On the other hand, rather innovative but contradictory, later findings for GlyT2 by the same group suggested that ubiquitination of four *C*-terminus lysine residues is required for constitutive endocytosis and only combined mutations to arginine abolished constitutive endocytosis [42].

The contradictory results between redundancy and specificity in the mechanism of GlyT1 ubiquitination can be explained by the following reasons: (i) In the present work, we report ubiquitination analysis using the mouse GlyT1a and human GlyT1b and c isoforms, whereas the group of Fernandez-Sanchez *et al*. (2009) performed the analysis using the rat GlyT1b isoform. Interestingly, the rat and human isoforms share the same number and position of lysine residues at the C-terminus; (2) Fernandez-Sanchez *et al*. used transient transfection of MDCK cells to express GlyT1b; by contrast, we used PAE cell lines that stably express GlyT1. In our experience, the studies on DAT and GlyT transporter ubiquitination suggest that only a minor portion of the total transporter can be detected as ubiquitinated, suggesting that large quantities of purified protein should be analyzed to detect consistent differences in the levels of ubiquitination. Stable cell lines offer the advantage of a more homogeneous cell population where the majority of the cells express transporter at the plasma membrane and allow the assays to be performed with larger quantities of transporter protein. This is compared to transient transfection assays, where expression is more heterogeneous and many cells can remain untransfected, in addition to lower protein yields.

Multiple reports support our hypothesis of redundancy and multiple choices of lysine residue used for modification. Consistent with our results, abolishment of ubiquitination for the glutamate transporter GLT1 required a total of 11 mutations, corresponding to all *N*- and *C*-terminus lysines. Similar findings were obtained for the choline transporter CHT1, which required 10 substitutions of the 12 intracellular lysine residues to reduce the levels of ubiquitination [13,43]. The classical example of redundancy is provided by the epidermal growth factor receptor (EGFR), where substitution of a minimum of six lysines in the kinase domain resulted in a dramatic decrease in the overall ubiquitination, but it was still not completely abolished [44]. Interestingly, these authors reported that receptor ubiquitination was responsible for targeting to the lysosome but did not affect ligand-induced internalization. Finally, in a recent report on the hedgehog 7-transmembrane domain receptor Smo1 from *Drosophila*, the authors demonstrated that only replacement of all 48 cytosolic lysines to arginine abolished ubiquitination and the mutant largely accumulated on the cell surface, suggesting defective endocytosis [45]. Altogether, these findings demonstrated the lack of preference in lysine selection during the mechanism of GlyT1 transporter ubiquitination and its implication in glycine capacity, endocytosis and intracellular sorting to the lysosome.

Although the majority of data describing transporter postranslation modifications have been obtained by expressing the transporter in model cells, the components of such mechanisms are beginning to be elucidated in the brain. For example, a physical association of PKCβ with DAT has been shown by co-immunoprecipitation from rat striatal synaptosomes [46], suggesting a direct interaction of both proteins. In addition, a dramatic reduction in dopamine efflux was observed in striatal synaptosomes from a PKCβ knockout mice, providing additional evidence of PKCβ as the potential kinase that phosphorylate transporter and induces substrate efflux[47]. Consistent with these findings, in heterologous models we have observed abolishment of GlyT1 phosphorylation by pharmacological inhibition of PKCβ, pointing to this kinase as responsible for phosphorylation [10]. [48,49,50] By contrast, to demonstrate PKC-dependent transporter ubiquitination in neurons has been technically difficult, in part due to low levels of total transporter immunoprecipitated from cultured neurons or brain slices. In addition, the low levels of ubiquitinated transporters recovered after PKC activation from heterologous systems suggest that large quantities of transporter must be purified in order to observe the signal of ubiquitinated transporters.

For GlyTs, the lack of knowledge about the precise location of glycinergic circuits has complicated the analysis of many mechanisms with the endogenous transporters. However, based on findings described in this manuscript for GlyT1 and other family members we can speculate about the physiological relevance of these modifications in the CNS. It has been shown for the close relative, the dopamine transporter, that the pool of endogenous transporter is distributed at the cell surface and in EEA1-, Rab7- and Rab11-positive endosomes, showing the dynamics between endocytosis, recycling and sorting of transporter to late endosomes in neurons [51,52]. Moreover, PKC activation promotes DAT endocytosis above constitutive levels, leading to a transient or sustained inhibition [53,54]. These results support a model whereby depolarization-dependent Ca^2+^ influx will trigger fast synaptic vesicle fusion and neurotransmitter release, accompanied by Ca^2+^-dependent activation of conventional PKC. Activated PKC could directly or indirectly lead to phosphorylation of the E3 ligase Nedd4, which has been shown to be involved in transporter ubiquitination, ultimately increasing the affinity for transporter and resulting in transporter ubiquitination and enhanced endocytosis. Nedd4-2 phosphorylation has been shown to increase its affinity for TrkA and promote receptor ubiquitination [55]. Transporter endocytosis could prevent neurotransmitter re-uptake to allow activation of postsynaptic receptors. Further de-ubiquitination and transporter recycling back to the cell surface will make possible re-uptake of neurotransmitter and termination of neurotransmission. However, future analysis of the endogenous transporter will shed light into the direct role of these posttranslational modifications on trafficking and glycinergic neurotransmission.

## Supporting Information

S1 Fig PKC-dependent GlyT1 degradation. (PDF)

## Abbreviations

GlyT: glycine transporter
EEA1: early endosome antigen 1
PAE: porcine aortic endothelial cells
PKC: protein kinase C
PMA: Phorbol 12-myristate 13-acetate
YFP: yellow fluorescent protein
WT: wild-type

## References

[1] Johnson JW, Ascher P (1987) Glycine potentiates the NMDA response in cultured mouse brain neurons. Nature 325: 529–531. 2433595 10.1038/325529a0

[2] Imamura Y, Ma CL, Mohan P, Bergeron R (2008) Sustained saturating level of glycine induces changes in NR2B-containing-NMDA receptor localization in the CA1 region of the hippocampus. Journal of Neurochemistry 105: 2454–2465. 10.1111/j.1471-4159.2008.05324.x 18331477

[3] Bergeron R, Meyer TM, Coyle JT, Greene RW (1998) Modulation of N-methyl-D-aspartate receptor function by glycine transport. PNAS 95: 15730–15734. 9861038 10.1073/pnas.95.26.15730 PMC28112

[4] Supplisson S, Bergman C (1997) Control of NMDA receptor activation by a glycine transporter co-expressed in Xenopus oocytes. J Neurosci 17: 4580–4590. 9169519 10.1523/JNEUROSCI.17-12-04580.1997 PMC6573333

[5] Cubelos B, Gimenez C, Zafra F (2005) Localization of the GLYT1 Glycine Transporter at Glutamatergic Synapses in the Rat Brain. Cereb Cortex 15: 448–459. 15749988 10.1093/cercor/bhh147

[6] Jursky F, Nelson N (1996) Developmental Expression of the Glycine Transporters GLYT1 and GLYT2 in Mouse Brain. Journal of Neurochemistry 67: 336–344. 8667011 10.1046/j.1471-4159.1996.67010336.x

[7] Hanley JG, Jones EMC, Moss SJ (2000) GABA Receptor rho 1 Subunit Interacts with a Novel Splice Variant of the Glycine Transporter, GLYT-1. J Biol Chem 275: 840–846. 10625616 10.1074/jbc.275.2.840

[8] Geerlings A, Lopez-Corcuera B, Aragon C (2000) Characterization of the interactions between the glycine transporters GLYT1 and GLYT2 and the SNARE protein syntaxin 1A. FEBS Lett 470: 51–54. 10722844 10.1016/s0014-5793(00)01297-7

[9] Sung U, Apparsundaram S, Galli A, Kahlig KM, Savchenko V, Schroeter S, et al (2003) A Regulated Interaction of Syntaxin 1A with the Antidepressant-Sensitive Norepinephrine Transporter Establishes Catecholamine Clearance Capacity. J Neurosci 23: 1697–1709. 12629174 10.1523/JNEUROSCI.23-05-01697.2003 PMC6741950

[10] Vargas-Medrano J, Castrejon-Tellez V, Plenge F, Ramirez I, Miranda M (2011) PKCbeta-dependent phosphorylation of the glycine transporter 1. Neurochem Int 59: 1123–1132. 10.1016/j.neuint.2011.08.006 21864610 PMC3226844

[11] Miranda M, Wu CC, Sorkina T, Korstjens DR, Sorkin A (2005) Enhanced Ubiquitylation and Accelerated Degradation of the Dopamine Transporter Mediated by Protein Kinase C. J Biol Chem 280: 35617–35624. 16109712 10.1074/jbc.M506618200

[12] Miranda M, Dionne KR, Sorkina T, Sorkin A (2007) Three Ubiquitin Conjugation Sites in the Amino Terminus of the Dopamine Transporter Mediate Protein Kinase C-dependent Endocytosis of the Transporter. Mol Biol Cell 18: 313–323. 17079728 10.1091/mbc.E06-08-0704 PMC1751334

[13] Sheldon AL, Gonzalez MI, Krizman-Genda EN, Susarla BT, Robinson MB (2008) Ubiquitination-mediated internalization and degradation of the astroglial glutamate transporter, GLT-1. Neurochem Int 53: 296–308. 10.1016/j.neuint.2008.07.010 18805448 PMC2629405

[14] Geerlings A, Nunez E, Lopez-Corcuera B, Aragon C (2001) Calcium- and Syntaxin 1-mediated Trafficking of the Neuronal Glycine Transporter GLYT2. J Biol Chem 276: 17584–17590. 11278707 10.1074/jbc.M010602200

[15] Horiuchi M, Loebrich S, Brandstaetter JH, Kneussel M, Betz H (2005) Cellular localization and subcellular distribution of Unc-33-like protein 6, a brain-specific protein of the collapsin response mediator protein family that interacts with the neuronal glycine transporter 2. J Neurochem 94: 307–315. 15998282 10.1111/j.1471-4159.2005.03198.x

[16] Ohno K, Koroll M, El Far O, Scholze P, Gomeza J, Betz H (2004) The neuronal glycine transporter 2 interacts with the PDZ domain protein syntenin-1. Mol Cell Neurosci 26: 518–529. 15276154 10.1016/j.mcn.2004.04.007

[17] Foster JD, Pananusorn B, Vaughan RA (2002) Dopamine Transporters Are Phosphorylated on N-terminal Serines in Rat Striatum. J Biol Chem 277: 25178–25186. 11994276 10.1074/jbc.M200294200

[18] Granas C, Ferrer J, Loland CJ, Javitch JA, Gether U (2003) N-terminal Truncation of the Dopamine Transporter Abolishes Phorbol Ester- and Substance P Receptor-stimulated Phosphorylation without Impairing Transporter Internalization. J Biol Chem 278: 4990–5000. 12464618 10.1074/jbc.M205058200

[19] Gomez LL, Alam S, Smith KE, Horne E, Dell'Acqua ML (2002) Regulation of A-Kinase Anchoring Protein 79/150-cAMP-Dependent Protein Kinase Postsynaptic Targeting by NMDA Receptor Activation of Calcineurin and Remodeling of Dendritic Actin. J Neurosci 22: 7027–7044. 12177200 10.1523/JNEUROSCI.22-16-07027.2002 PMC6757891

[20] Gant VU Jr, Moreno S, Varela-Ramirez A, Johnson KL (2014) Two membrane-associated regions within the Nodamura virus RNA-dependent RNA polymerase are critical for both mitochondrial localization and RNA replication. J Virol 88: 5912–5926. 10.1128/JVI.03032-13 24696464 PMC4093860

[21] Sorkina T, Doolen S, Galperin E, Zahniser NR, Sorkin A (2003) Oligomerization of Dopamine Transporters Visualized in Living Cells by Fluorescence Resonance Energy Transfer Microscopy. J Biol Chem 278: 28274–28283. 12746456 10.1074/jbc.M210652200

[22] Sato K, Adams R, Betz H, Schloss P (1995) Modulation of a recombinant glycine transporter (GLYT1b) by activation of protein kinase C. J Neurochem 65: 1967–1973. 7595479 10.1046/j.1471-4159.1995.65051967.x

[23] Bradford MM (1976) A rapid and sensitive method for the quantitation of microgram quantities of protein utilizing the principle of protein-dye binding. Anal Biochem 72: 248–254. 942051 10.1016/0003-2697(76)90527-3

[24] Fernandez-Sanchez E, Martinez-Villarreal J, Gimenez C, Zafra F (2009) Constitutive and regulated endocytosis of the glycine transporter GLYT1b is controlled by ubiquitination. J Biol Chem 284: 19482–19492. 10.1074/jbc.M109.005165 19473961 PMC2740574

[25] Sorkina T, Miranda M, Dionne KR, Hoover BR, Zahniser NR, Sorkin A (2006) RNA Interference Screen Reveals an Essential Role of Nedd4-2 in Dopamine Transporter Ubiquitination and Endocytosis. J Neurosci 26: 8195–8205. 16885233 10.1523/JNEUROSCI.1301-06.2006 PMC6673793

[26] Mortensen OV, Larsen MB, Prasad BM, Amara SG (2008) Genetic complementation screen identifies a mitogen-activated protein kinase phosphatase, MKP3, as a regulator of dopamine transporter trafficking. Mol Biol Cell 19: 2818–2829. 10.1091/mbc.E07-09-0980 18434601 PMC2441689

[27] Zafra F, Aragon C, Olivares L, Danbolt NC, Gimenez C, Storm-Mathisen J (1995) Glycine transporters are differentially expressed among CNS cells. J Neurosci 15: 3952–3969. 7751957 10.1523/JNEUROSCI.15-05-03952.1995 PMC6578198

[28] Mohler H, Boison D, Singer P, Feldon J, Pauly-Evers M, Yee BK (2011) Glycine transporter 1 as a potential therapeutic target for schizophrenia-related symptoms: evidence from genetically modified mouse models and pharmacological inhibition. Biochem Pharmacol 81: 1065–1077. 10.1016/j.bcp.2011.02.003 21333635

[29] Sassoe-Pognetto M, Wassle H, Grunert U (1994) Glycinergic synapses in the rod pathway of the rat retina: cone bipolar cells express the alpha 1 subunit of the glycine receptor. J Neurosci 14: 5131–5146. 8046473 10.1523/JNEUROSCI.14-08-05131.1994 PMC6577182

[30] Wassle H, Heinze L, Ivanova E, Majumdar S, Weiss J, Harvey RJ, et al (2009) Glycinergic transmission in the Mammalian retina. Front Mol Neurosci 2: 6 10.3389/neuro.02.006.2009 19924257 PMC2777502

[31] Eriksen J, Bjorn-Yoshimoto WE, Jorgensen TN, Newman AH, Gether U (2010) Postendocytic sorting of constitutively internalized dopamine transporter in cell lines and dopaminergic neurons. J Biol Chem 285: 27289–27301. 10.1074/jbc.M110.131003 20551317 PMC2930728

[32] Susarla BT, Robinson MB (2008) Internalization and degradation of the glutamate transporter GLT-1 in response to phorbol ester. Neurochem Int 52: 709–722. 17919781 10.1016/j.neuint.2007.08.020 PMC2292111

[33] Moritz AE, Foster JD, Gorentla BK, Mazei-Robison MS, Yang J-W, Sitte HH, et al (2013) Phosphorylation of Dopamine Transporter Serine 7 Modulates Cocaine Analog Binding. Journal of Biological Chemistry 288: 20–32. 10.1074/jbc.M112.407874 23161550 PMC3537014

[34] Foster JD, Yang J-W, Moritz AE, ChallaSivaKanaka S, Smith MA, Holy M, et al (2012) Dopamine Transporter Phosphorylation Site Threonine 53 Regulates Substrate Reuptake and Amphetamine-stimulated Efflux. Journal of Biological Chemistry 287: 29702–29712. 10.1074/jbc.M112.367706 22722938 PMC3436161

[35] Garcia-Tardon N, Gonzalez-Gonzalez IM, Martinez-Villarreal J, Fernandez-Sanchez E, Gimenez C, Zafra F (2012) Protein kinase C (PKC)-promoted endocytosis of glutamate transporter GLT-1 requires ubiquitin ligase Nedd4-2-dependent ubiquitination but not phosphorylation. J Biol Chem 287: 19177–19187. 10.1074/jbc.M112.355909 22505712 PMC3365950

[36] Gonzalez-Gonzalez IM, Garcia-Tardon N, Gimenez C, Zafra F (2008) PKC-dependent endocytosis of the GLT1 glutamate transporter depends on ubiquitylation of lysines located in a C-terminal cluster. Glia 56: 963–974. 10.1002/glia.20670 18381652

[37] Nissen-Meyer LS, Popescu MC, Hamdani el H, Chaudhry FA (2011) Protein kinase C-mediated phosphorylation of a single serine residue on the rat glial glutamine transporter SN1 governs its membrane trafficking. J Neurosci 31: 6565–6575. 10.1523/JNEUROSCI.3694-10.2011 21525297 PMC6622677

[38] Gorentla BK, Moritz AE, Foster JD, Vaughan RA (2009) Proline-directed phosphorylation of the dopamine transporter N-terminal domain. Biochemistry 48: 1067–1076. 10.1021/bi801696n 19146407 PMC2668220

[39] Kalandadze A, Wu Y, Robinson MB (2002) Protein kinase C activation decreases cell surface expression of the GLT-1 subtype of glutamate transporter. Requirement of a carboxyl-terminal domain and partial dependence on serine 486. J Biol Chem 277: 45741–45750. 12324450 10.1074/jbc.M203771200

[40] de Juan-Sanz J, Zafra F, Lopez-Corcuera B, Aragon C (2011) Endocytosis of the Neuronal Glycine Transporter GLYT2. Role of Membrane Rafts and Protein Kinase C-dependent Ubiquitination. Traffic. 10.1111/j.1600-0854.2011.01278.x 21910806

[41] Fornes A, Nunez E, Aragon C, Lopez-Corcuera B (2004) The second intracellular loop of the glycine transporter 2 contains crucial residues for glycine transport and phorbol ester-induced regulation. J Biol Chem 279: 22934–22943. 15010455 10.1074/jbc.M401337200

[42] de Juan-Sanz J, Nunez E, Lopez-Corcuera B, Aragon C (2013) Constitutive endocytosis and turnover of the neuronal glycine transporter GlyT2 is dependent on ubiquitination of a C-terminal lysine cluster. PLoS One 8: e58863 10.1371/journal.pone.0058863 23484054 PMC3590132

[43] Yamada H, Imajoh-Ohmi S, Haga T (2012) The high-affinity choline transporter CHT1 is regulated by the ubiquitin ligase Nedd4-2. Biomed Res 33: 1–8. 22361880 10.2220/biomedres.33.1

[44] Huang F, Kirkpatrick D, Jiang X, Gygi S, Sorkin A (2006) Differential regulation of EGF receptor internalization and degradation by multiubiquitination within the kinase domain. Mol Cell 21: 737–748. 16543144 10.1016/j.molcel.2006.02.018

[45] Yang X, Mao F, Lv X, Zhang Z, Fu L, Lu Y, et al (2013) Drosophila Vps36 regulates Smo trafficking in Hedgehog signaling. J Cell Sci 126: 4230–4238. 10.1242/jcs.128603 23843610

[46] Johnson LA, Guptaroy B, Lund D, Shamban S, Gnegy ME (2005) Regulation of amphetamine-stimulated dopamine efflux by protein kinase C beta. J Biol Chem 280: 10914–10919. 15647254 10.1074/jbc.M413887200

[47] Chen R, Furman CA, Zhang M, Kim MN, Gereau RW 4th, Leitges M, et al (2009) Protein kinase Cbeta is a critical regulator of dopamine transporter trafficking and regulates the behavioral response to amphetamine in mice. J Pharmacol Exp Ther 328: 912–920. 10.1124/jpet.108.147959 19098163 PMC2682265

[48] Carvelli L, Moron JA, Kahlig KM, Ferrer JV, Sen N, Lechleiter JD, et al (2002) PI 3-kinase regulation of dopamine uptake. J Neurochem 81: 859–869. 12065645 10.1046/j.1471-4159.2002.00892.x

[49] Fog JU, Khoshbouei H, Holy M, Owens WA, Vaegter CB, Sen N, et al (2006) Calmodulin kinase II interacts with the dopamine transporter C terminus to regulate amphetamine-induced reverse transport. Neuron 51: 417–429. 16908408 10.1016/j.neuron.2006.06.028

[50] Dipace C, Sung U, Binda F, Blakely RD, Galli A (2007) Amphetamine induces a calcium/calmodulin-dependent protein kinase II-dependent reduction in norepinephrine transporter surface expression linked to changes in syntaxin 1A/transporter complexes. Mol Pharmacol 71: 230–239. 17032905 10.1124/mol.106.026690

[51] Rao A, Simmons D, Sorkin A (2011) Differential subcellular distribution of endosomal compartments and the dopamine transporter in dopaminergic neurons. Mol Cell Neurosci 46: 148–158. 10.1016/j.mcn.2010.08.016 20816972 PMC3018570

[52] Eriksen J, Jorgensen TN, Gether U (2010) Regulation of dopamine transporter function by protein-protein interactions: new discoveries and methodological challenges. J Neurochem 113: 27–41. 10.1111/j.1471-4159.2010.06599.x 20085610

[53] Hong WC, Amara SG (2013) Differential targeting of the dopamine transporter to recycling or degradative pathways during amphetamine- or PKC-regulated endocytosis in dopamine neurons. FASEB J 27: 2995–3007. 10.1096/fj.12-218727 23612789 PMC3714572

[54] Gabriel LR, Wu S, Kearney P, Bellve KD, Standley C, Fogarty KE, et al (2013) Dopamine transporter endocytic trafficking in striatal dopaminergic neurons: differential dependence on dynamin and the actin cytoskeleton. J Neurosci 33: 17836–17846. 10.1523/JNEUROSCI.3284-13.2013 24198373 PMC3818556

[55] Arevalo JC, Waite J, Rajagopal R, Beyna M, Chen ZY, Lee FS, et al (2006) Cell survival through Trk neurotrophin receptors is differentially regulated by ubiquitination. Neuron 50: 549–559. 16701206 10.1016/j.neuron.2006.03.044

