# Supplementary material for: PKC-Dependent GlyT1 Ubiquitination Occurs Independent of Phosphorylation: Inespecificity in Lysine Selection for Ubiquitination
Source: PLoS One. 2015 Sep 29;10(9):e0138897. doi: 10.1371/journal.pone.0138897 (PMC4587969; doi:10.1371/journal.pone.0138897)
Supplement: S1 Fig — (PDF) [file pone.0138897.s001.pdf]

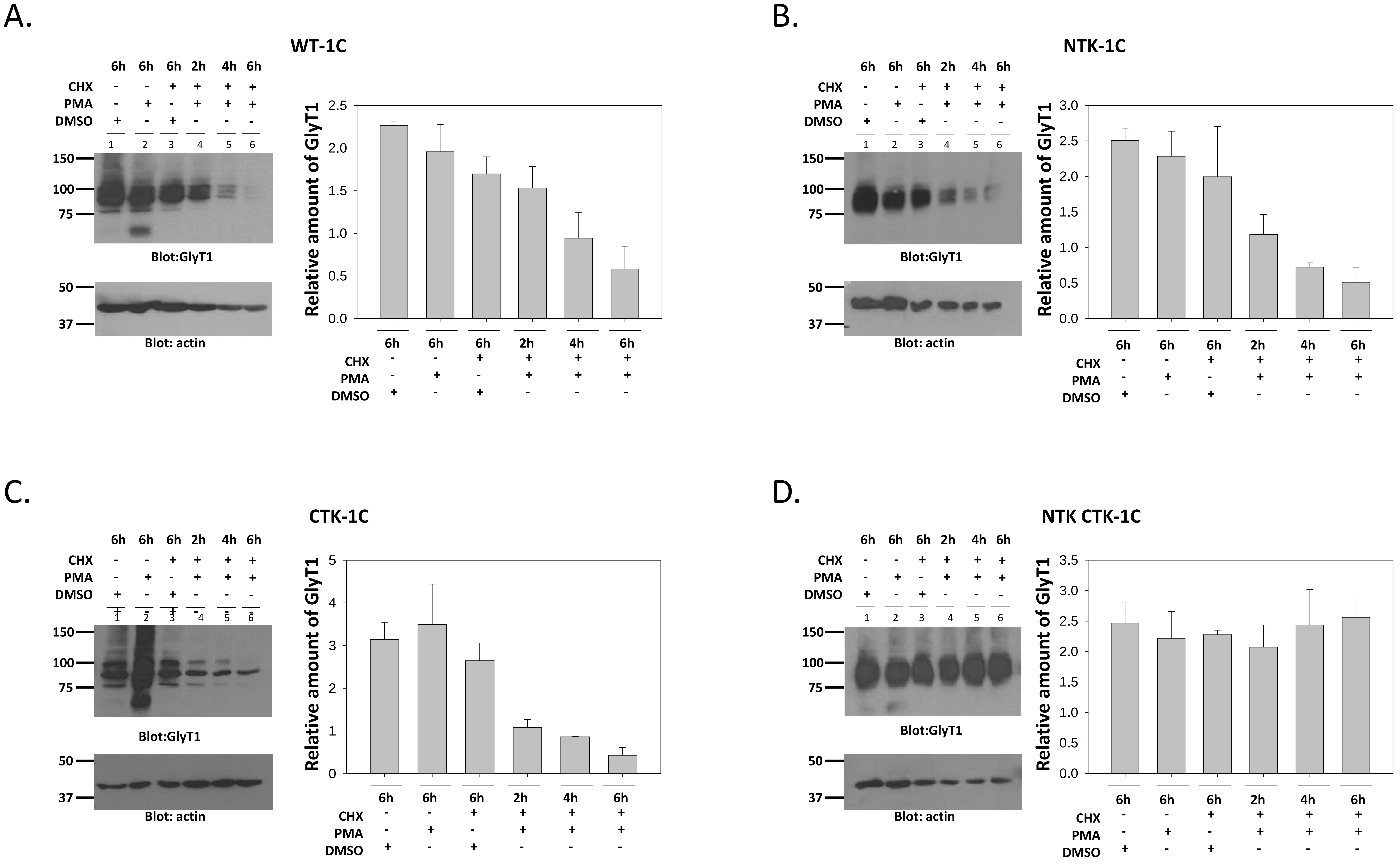
A.
WT-1C
B.
NTK-1C
C.
CTK-1C
D.
NTK CTK-1C
6h 6h 6h 2h 4h 6h
CHX - - + + + +
PMA - + - + + +
DMSO + - + - - -
150
100
75
50
37
Blot:GlyT1
Blot: actin
Relative amount of GlyT1

Supplemental Figure S1. PKC-dependent GlyT1 degradation. PAE cells expressing WT and mutant forms of GlyT1were incubated with 50µg/ml of cycloheximide (CHX) for 2h followed by treatment with 1µM PMA for 0-6 hr. After incubation with PMA, all cells were incubated in the presence of CHX for a total of 8 h. In control experiments (lanes 1 and 2), cells were incubated with DMSO or PMA for 6 h in the absence of CHX. After incubations, the cells were lysed and total lysates subjected to SDS-PAGE and western blot with GlyT1 and actin antibodies. A) WT-FH-GlyT1c, and mutants B) NTK-1c, C) CTK-1c, and D) NTK-CTK-1c. Densitometry values are expressed as a mean $\pm$ SEM, n=2-3.
